# Complete Genome Sequence of a Wild-Type Isolate of Caulobacter vibrioides Strain CB1

**DOI:** 10.1128/MRA.01153-18

**Published:** 2018-09-27

**Authors:** Derrick C. Scott, Kiesha Wilson, Keshawn Ross, Damyen Ingram, Tajah Lewter, Jasmine Herring, David Duncan, Anthea Aikins, Bert Ely

**Affiliations:** a Department of Biological Sciences, Delaware State University, Dover, Delaware, USA; b Department of Biological Sciences, University of South Carolina, Columbia, South Carolina, USA; University of Maryland School of Medicine

## Abstract

The complete genome sequence of Caulobacter vibrioides strain CB1 consists of a chromosome of 4,137,285 bp, with a GC content of 67.2% and 3,990 coding DNA sequences. This strain contains the typical genome rearrangement that is characteristic of the Caulobacter strains that are currently sequenced. However, this strain is so closely related to sequenced strain NA1000 that rearrangements were minimal. This will allow further clarification of the causes of rearrangements in the species.

## Abstract (precis)

The complete genome sequence of Caulobacter vibrioides strain CB1 consists of a chromosome of 4,137,285 bp, with a GC content of 67.2% and 3,990 coding DNA sequences. This strain contains the typical genome rearrangement that is characteristic of the Caulobacter strains that are currently sequenced.

## ANNOUNCEMENT

When the genome sequences of Caulobacter isolates NA1000 and K31 were compared, numerous genome rearrangements were observed (1). This phenomenon was dubbed genome scrambling. In contrast, similar comparisons of closely related species of other bacterial genera revealed nominal rearrangements. A phylogenetic analysis of the genomic sequences of additional Caulobacter strains has revealed insight into the mechanisms of genome scrambling, but more genomic sequences are needed to gain additional clarity. Here, we report the sequence of the total genomic material of an additional C. vibrioides wild-type strain, CB1, which was sampled from tap water in California.

The strain C. vibrioides CB1 was isolated from tap water in California (2). The cells were cultivated at 30°C for 48 h in peptone yeast extract medium (3), which contained 2 g Bacto peptone, 1 g yeast extract, 0.5 M MgSO_4_, and 0.5 M CaCl_2_ per liter. Genomic DNA was isolated using the Qiagen DNeasy tissue kit following the manufacturer’s protocol. The primers 16S_533F (GTGCCAGCMGCCGCGGTAA) and 16S_U1492R (GGTTACCTTGTTACGACTT) were used to amplify the 16S rRNA region of the genome, and the amplified DNA was sequenced using Sanger technology on an ABI 3730 sequencer (GENEWIZ, USA). Genomic DNA sequencing was performed by the Delaware Bioinformatics Institute using a PacBio RS II sequencer, as suggested in previous studies for bacterial genomes with high GC contents (4). The resulting genome sequence was assembled using the Hierarchical Genome Assembly Process (HGAP) (5) in SMRT Portal through Amazon Machine Image (AMI) EC2 using the smrtanalysis-2.3.0-ami-20fb4848 image with the default *de novo* parameters. The sequence was annotated using the RAST server (http://rast.nmpdr.org) and the NCBI Prokaryotic Genome Annotation Pipeline (6–8) and then visualized and edited in Artemis (9).

The C. vibrioides CB1 complete genome consists of a circular chromosome of 4,137,285 bp, with a GC content of 67.2%. The genome is predicted to contain 3,990 coding sequences (CDSs). The numbers of tRNAs and rRNA operons are 51 and 2, respectively.

C. vibrioides CB1 has no inversions and only one insertion when compared to the closely related NA1000 strain. This is the first instance where two Caulobacter strains have revealed so few rearrangements when directly compared. Further studies between these strains will shed light into the mechanisms of genome rearrangement in Caulobacter species.

### Data availability.

The complete genome sequence of C. vibrioides CB1 has been deposited in GenBank under the accession number CP023314. The raw reads are also available under SRA accession number SRP158680.
